# High Dose Infliximab in the Treatment of Refractory Uveitis: Does Dose Matter?

**DOI:** 10.5402/2012/765380

**Published:** 2012-01-05

**Authors:** Sukesh Sukumaran, Katherine Marzan, Bracha Shaham, Andreas Reiff

**Affiliations:** Division of Pediatric Rheumatology, Children's Hospital Los Angeles, Los Angeles, CA 90027, USA

## Abstract

*Background*. Infliximab (INF) has been shown to be beneficial in treating refractory uveitis, however, no data exist on optimal dosing and the efficacy of higher dosing. *Objectives*. To compare the efficacy of low-dose (LD) (<10 mg/kg), moderate-dose (MD) (≥10–15 mg/kg), and high-dose (HD) INF (≥15–20 mg/kg) in the treatment of uveitis. *Methods*. Retrospective chart review children with uveitis diagnosed at Childrens Hospital Los Angeles and Millers Children's Hospital, CA, USA. *Results*. Of the 34 INF-treated children, 6 patients received LD, 19 received MD, and 9 received HD. Average disease duration prior to therapy was 10.6, 24.6, and 37.1 months each group, respectively. Topical steroids were discontinued after an average of 3 months, 9.5 months, and 10.2 months in the LD, MD, and HD groups, respectively. We found that 66% of patients receiving LD, 42% of MD, and 66% receiving HD INF failed therapy and required either dose escalation or alternate medication for disease control. *Conclusions*. INF is beneficial in the treatment of uveitis, and dose escalation up to 4 times above the approved dose is often necessary to achieve disease control in patients with uveitis. Doses < 10 mg/kg every 4 weeks may not be sufficient to control disease.

## 1. Introduction

The term uveitis defines several ocular disease states characterized by inflammation of the entire or individual parts of the uveal tract including iris, ciliary body, or choroid. Uveitis can be classified by location in the eye as anterior uveitis or iridocyclitis, intermediate uveitis or pars planitis, and posterior uveitis or by primary and secondary uveitis, whereby primary uveitis refers to intraocular inflammation of unknown etiology, and secondary uveitis refers to inflammatory ocular conditions that are either associated with systemic, intrinsic, or infectious diseases. Uveitis is the most common ocular manifestation of childhood rheumatic diseases such as juvenile idiopathic arthritis (JIA) and is the third most common preventable cause of blindness in the developed world [1, 2]. The risk of blindness is higher in children compared to the adult population mostly because of the higher rate of posterior uveitis [3]. 

The etiology and pathogenesis of uveitis is not well established, and multiple theories exist. Animal model data suggest that autoimmune uveitis is driven by a predominant T helper-1 (Th-1) response of autoreactive T cells [4, 5]. Th1 cells produce the cytokines such as interleukin- (IL-) 2, IL-12, and IL-18 [6], while cell-mediated immunity is mainly associated with tumor necrosis factor (TNF)-*α* [7, 8]. Intravitreal injection of TNF-*α* in rabbits [9] and in rats [10] induces acute uveitis characterized by an increase in flare (aqueous humor protein) and a polymorphonuclear infiltrate in the anterior chamber, suggesting that TNF-*α* may be an initiating factor in the pathogenesis of uveitis. Elevated levels of IL-1*β* and TNF-*α* in the vitreous and serum of patients with uveitis suggests that uveitis is driven by a systemic inflammatory response [11]. It has been postulated that both TNF-*α* and IL-1*β* in conjunction with vascular endothelial growth factor (VEGF) lead to increased vascular permeability and damage in the blood retinal barrier (BRB) [12, 13]. The damage in the BRB leads to increase in transendothelial transport activity and exposure of the inner eye to the immune attack [14]. IL-6 and IL-1 may act as local amplification signals in chronic eye inflammation after the BRB has been breached. Other proinflammatory cytokines such as IL-2, IL4, IL-8, IL-12, IL-15, and IL17 along with chemokines such as metalloproteinases play an important role in the maintenance of chronic inflammation of the eye [15–17]. It has been shown that different cytokines dominate in different types of autoimmune uveitis. IL-1 and TNF-*α* are predominant cytokines in anterior uveitis [18], while IL-6, IL-12, and IL-23 are predominant in the Vogt-Koyanagi-Harada (VKH) syndrome [19, 20]. IL-6 and IL-12 are the predominant cytokines in Behcet's syndrome [21].

A variety of immunomodulatory agents are used in the management of uveitis. These include among others methotrexate, cyclosporine, and mycophenolate mofetil [22–24]. Anti-TNF agents like etanercept (ETN), adalimumab, and INF have been successfully used in the management of treatment refractory uveitis and several retrospective case series describe the use of INF in pediatric uveitis [25, 26]. 

Comparisons between INF and ETN in the treatment of uveitis have also been well described in the literature [27]. Saurenmann et al. retrospectively studied 21 children treated with ETN [11] and INF [13]. They concluded that patients receiving INF had a better response as compared with those receiving ETN (*P* = 0.048). Tynjälä et al. retrospectively reviewed children on either INF or ETN and concluded that patients receiving INF had more improvement in uveitis activity and a reduced number of flares per year (*P* = 0.015) compared to ETN. An international cross-sectional survey of pediatric rheumatologists was done by Foeldvari and Wierk. and showed that INF was found to be more efficacious; 70% of INF patients showed a good response compared to 53% of patients on ETN. Vazquez-Cobian et al. conducted a prospective study of adalimumab in pediatric uveitis (*n* = 14) and showed decreased inflammation in 13/14 patients and with a sustained response for 18 months after initiation of therapy [28]. A retrospective study by Biester et al. evaluated adalimumab in refractory uveitis (*n* = 18) and showed that sixteen patients had a good response, 15 of which were able to stop steroid therapy [29].

INF is a chimeric monoclonal antibody that binds to and inhibits tumor necrosis factor alpha, a cytokine that plays an important role in a variety of inflammatory processes, including induction of proinflammatory cytokines, enhancement of leukocyte migration, activation of neutrophil and eosinophil activity, induction of acute phase reactants and other liver proteins, and tissue degrading enzymes produced by synoviocytes and chondrocytes [30–32]. Initial studies with INF in uveitis secondary to Behcet's disease used traditional arthritis doses of INF [33]. Studies done in rabbit eyes show that high dose (20 mg/kg) of INF successfully preventing endotoxin-induced uveitis [34]. Kahn et al. did a retrospective study on 17 patients with chronic noninfectious uveitis who were treated with INF in high doses 10–20 mg/kg with a favorable outcome [30, 33]. However, no data exist on optimal dosing regimens in children, and the true efficacy of higher dosing remains unclear.

The objective of our study was to compare the efficacy of low-dose (LD) at <10 mg/kg IV, moderate-dose (MD) at ≥10 mg/kg to 15 mg/kg IV, and high-dose INF (HD) at ≥15 mg/kg–20 mg/kg IV given monthly in the treatment of various forms of uveitis.

## 2. Patients and Methods

This study was conducted at the Pediatric Rheumatology Core at Children's Hospital Los Angeles (CHLA) and Miller Children's Hospital Long Beach (MCH) from November, 2008 to December, 2009. The study population included children ≤21 years of age with uveitis who received INF therapy. We identified 34 children with various forms of uveitis of whom received INF for greater than one year and were included in the study. 

We performed a comprehensive medical record review using a standardized instrument to identify demographic information and clinical characteristics of the disease. We also collected age, gender, ocular and systemic diagnosis, previous and current medications, duration of each medication, and markers of inflammation and ocular inflammatory parameters. We documented dosage of INF administered and assigned patients to three groups depending on the INF dosage they received. Patients who received INF at <10 mg/kg given IV every four weeks were categorized as low dose (LD). Those who received INF given ≥10 to <15 mg/kg IV every four weeks were categorized under moderate dose (MD), and those who received INF given every four weeks at ≥15 to 20 mg/kg IV were categorized into the high-dose (HD) group. Ophthalmological and laboratory parameters were recorded on these patients for one year after starting the INF therapy.

We defined uveitis and recorded disease activity using the standardization of uveitis nomenclature (SUN) grading scheme and terminology [35]. Accordingly, inactive disease is grade 0 cells, worsening activity is defined as two-step increases in the level of inflammation, improved activity is a two-step decrease in the level of inflammation, and remission is defined as inactive disease for ≥3 months after discontinuing all treatments for eye disease. The standardization of uveitis nomenclature (SUN) working group grading scheme [35] for anterior chamber cells and flare was also used to monitor the clinical improvement. In this system, the grading of the anterior chamber cells varies from 0 to 4+. This reflects cells in a field which is the size of 1 mm by 1 mm slit beam. According to the SUN working group grading for the cell count, grade 0 is <1 cells/field, grade 0.5 is 1–5 cell/field, grade 1+ is 6–15 cells/field, grade 2+ is 16–25 cells/field, grade 3+ is 26–50 cells/field, and grade 4+ is >50 cells/field. Flaring is graded as follows: grade 0 corresponds to no flare, grade 1+ is faint, grade 2+ is moderate flare with clear iris and lens details, grade 3+ is marked flare with hazy iris and lens details, and grade 4 is intense flare with fibrinous aqueous [35].

All patients who received INF were pre-medicated with acetaminophen (10–15 mg/kg) and diphenhydramine (0.5 mg/kg). Infusions were given initially every two weeks for the first two weeks and then every 4 weeks. All patients were monitored with routine laboratory analysis including complete blood count, comprehensive metabolic panel, erythrocyte sedimentation rate, C-reactive protein, and human antichimeric antibodies (HACA). Ophthalmological evaluations were done by several experienced pediatric ophthalmologists or pediatric uveitis specialists. Evaluations were performed every two to four weeks depending upon the severity of the disease activity. Ocular outcome was assessed by cell count in the anterior chamber (ACD), flare, intraocular pressure (IOP), improvement in visual acuity (VA), and ability to reduce or stop concomitant topical or systemic steroids.

## 3. Statistical Analysis

We performed descriptive analyses of the above. Testing of proportions was performed using Chi-squared or Fisher's exact test as appropriate. *P* values were considered significant if they were ≤0.05. When Spearman rank correlation was done with respect to dose and outcome, the Spearman rho was 0.123 with a *P* value of 0.67. We used STATA version 10 software to perform all calculations.

## 4. Results

Of the 34 children enrolled in the study, all patients had bilateral eye disease. Sixteen (47%) had anterior uveitis, 11 (32%) patients had panuveitis, five (15%) had posterior uveitis, and two (6%) had intermediate uveitis. The mean age at diagnosis of uveitis was 8.5 years (range 1–15 years). Of the 34 patients, 16 (47%) had idiopathic uveitis, 6 (18%) had uveitis associated with oligoarticular juvenile idiopathic arthritis (JIA), 3 (9%) had uveitis secondary to polyarticular JIA, 3 (9%) had uveitis associated with enthesitis-related arthritis, 2 (6%) had Vogt-Koyanagi-Harada disease (VKH), and one patient each had uveitis associated with chronic noninfectious osteomyelitis (CNO), sarcoidosis, Blau's syndrome, and psoriatic arthritis.  Table 1 demonstrates the characteristics of our study population. 

The mean age of the patients at the start of the INF was 11.23 years (range 2–20 years). At the initiation of INF, 62% (21/34) of patients were ≥10 years of age. The mean duration of uveitis prior to INF treatment was 2.62 years (2–8 years). The mean duration of steroid treatment prior to start of disease modifying antirheumatic drugs (DMARD) therapy was 2 years (0.6–4.6 years). Prior to INF therapy, all patients had been treated with steroids. This included 15/34 (44%) patients that received topical steroids and 19/34 (56%) patients that were on both topical and systemic steroids. The average dose of systemic steroid was 0.25 mg/kg, and the maximum dose was 20 mg per day for a mean period of 2.8 months. Topical prednisone administration varied significantly from every hour while awake to once a day. Based on the dosage given, there were 6 (18%) patients assigned to the LD group, 19 (56%) patients to the MD group, and 9 (26%) patients to the HD group. Dosing was determined by the primary pediatric rheumatologist on the basis of disease duration, disease activity at the time of presentation, and prior medication failures.

Overall, after six months of INF therapy, 25 (74%) patients had improvement in anterior chamber cell density, 27 (80%) had improvement in flare, and 20 (58.8%) had improved in visual acuity. We also found that 27 (80%) were able to wean topical steroids and 20 (58.8%) were able to discontinue topical steroids. However, there still remained 7/34 (21%) patients that were unable to wean the topical prednisone at six months of followup. Human antichimeric antibodies (HACA) were negative in 14/34 (41%) tested patients. These included 6 patients (67%) from the HD, 4 (21%) from the MD, and 4 (67%) from the LD groups. 

After one year of INF therapy, the anterior chamber cell density showed improvement in 67%, 68%, and 78% of patients in the LD, MD and HD groups, respectively. Improvement in flare was noted in 100%, 84% and 67% of the LD, MD, and HD, respectively. VA improved in 17%, 32%, and 56% in the LD, MD, and HD, respectively. The improvement in IOP was 11% in the MD, and 44% in the HD group. Topical or oral prednisone was weaned in 83% of patients in the LD, 74% in the MD, and 89% of the patients in the HD group.  Table 2 compares the ocular findings of patients at baseline and at 1 year of followup.

### 4.1. Low-Dose Group

Out of the 6 children in the LD group, one patient was started at 3 mg/kg and was advanced to 5 mg/kg after the first 3 months and continued on this dose for the remaining 9 months. Four of the 6 (67%) patients were started on 5 mg/kg but required escalation of dosage to 10 mg/kg due to lack of efficacy after 3.5 months. Only one patient who was started at 5 mg/kg that continued on the same dose during the entire observational period. 

Two patients were able to wean topical prednisone within a two-month period and were able to stop at 3 months. There was also improvement in ocular parameters noted in these 2 patients. The anterior cell chamber density (ACD) was 1.5 OU, while at 12 months, the ACD decreased to zero OU. Similarly, flare was 1.6 and 1.3 in left eye (OS) and right eye (OD) initially, and at the end of study period, the flare was zero OU. The remaining 4/6 patients were advanced to the MD group and subsequently were able to stoptopical prednisone after nine months. 

### 4.2. Moderate-Dose Group

Of the 19 patients in the MD group, 6 (31%) had anterior uveitis, while 13 (69%) had panuveitis, posterior uveitis, or intermediate uveitis. Eleven (58%) patients in the MD group improved significantly during the study period. The average time to discontinuation of steroids was 9.5 months (6–19 months) in this group. Among the 11 patients who were weaned off steroids and remained on MD of INF, ACD improved in 9 (81%) patients, and flare improved in 7 (64%) patients. Other markers like IOP improved in 2 (18%) children, and VA improved in 5 (45%) children. The mean ACD in OS and OD was 1.7 and 1.6 and decreased to 0.4 and 0.3 in OS and OD by one year. The mean flare was 1.8 and 1.6 in OS and OD at the start of therapy and improved to 0.05 and 0.15 OS and OD, respectively, by 12 in months. 

Eight (42%) children required a dose escalation after an average period of 7.5 month (range 6.5–9 months) secondary to worsening of the disease and required dosage escalation to HD group. All of the patients that were advanced to HD had nonanterior uveitis.

### 4.3. High-Dose Group

This group was distinct for the fact that patients had a higher percentage of nonanterior disease, a higher degree of chronicity, disease severity, and a higher rate of prior DMARD failures, which explains the use of initial higher dosing. Of the 9 patients in the HD group, 44% had anterior uveitis, and 55% had panuveitis, posterior uveitis, or intermediate uveitis. 

The mean ACD at the initiation of therapy in this group was 2.1 and 1.7 in OS and OD, respectively, and decreased significantly to 0.8 and 0.5 in OS and OD, respectively, by one year. Similarly, flare at the initiation of the treatment also decreased from 2.1 and 1.8 in OS and OD to 0.5 and 0.2 in OS and OD, respectively. Despite the use of high-dose INF, 7 (78%) patients were unable to wean topical prednisone at six months of INF therapy. Six other patients (67%) were changed to another class of medications including abatacept, rituximab, or daclizumab due to worsening of disease activity. One of these patients had been started on 15 mg/kg that required dose escalation to 20 mg/kg at 5.4 months due to inadequate response.

## 5. Discussion

Our study confirmed that treatment with INF is beneficial in the treatment of uveitis and dose escalation up to 4 times above the approved dose is often necessary to achieve disease control. Overall, 80% of patients had a good response to the INF treatment even though over 35% of the patients required a dose escalation to ≥10 mg/kg (4 from LD to MD and 8 from MD to HD) and 32/34 (94%) patients in this population received ≥10 mg/kg of INF at the end of the observation period. Treatment failure to the chosen dose occurred in 6/34 (18%) of patients, all of whom were in the HD group.

The justification of using very high doses at the initiation of therapy in our cohort was based on the characteristics of the patients encountered. Assuming that inflammatory eye disease follows the same rules as any other autoimmune disease, the longer patients are not receiving appropriate treatment, the more difficult to achieve a meaningful response late into the disease. In our study, the mean duration of uveitis prior to starting treatment was longer in the HD (37 months) and MD (25 months) group compared with LD (11 months) group. This may indicate that patients with prolonged duration of illness are less likely to respond to lower doses and longer dose intervals. As an additional poor prognostic factor, there were 11 patients with panuveitis, five with posterior uveitis, and two with intermediate uveitis in this group. These are traditionally more difficult diseases to control. As a result, visual acuity was also worse in the HD group at the initiation of the treatment compared to the LD group. Legal blindness (≥20/200) was present in 22 eyes. Similarly band keratopathy (BK) and keratic precipitates (KP) were seen in 15/56 (27%) eyes, and glaucoma was observed in 28/56 (50%) eyes.

 In the 10 patients who had anterior uveitis in the MD and HD group, the higher dose approach was chosen due to a very high level of acuity and severity in the initial ophthalmology exam. Although they responded very well to high dose of INF, their disease duration prior to initiation of INF was shorter than that in the other patients. This may suggest that an early and aggressive treatment approach may lead to better outcomes. Comparatively, the initial LD approach was utilized on those patients that had mostly anterior uveitis and less disease severity when compared with the HD or MD group. Two patients in the LD group achieved medicated remission by the third month. However, persistence of disease prompted four patients (67%) in the LD group to escalate dosing to the MD group at an average time of 3.5 months. The patients who were advanced to MD group subsequently did well and were able to stop prednisone after an average period of nine months. Eight (42%) patients in the MD group were advanced to the HD group due to the persistence of active disease and did well for the entire observational period. Six patients (67%) from the HD group were changed to other medications due to poor response in the first six months of treatment. The failure rate to the chosen INF dose in LD and HD was similar at 67%, while the MD had a lesser failure rate (42%). The negative HACA result in all the tested patients suggests that these antibodies had no influence on the lack of response to INF. 

We acknowledge our study has limitations. The limitations of this study are those inherent to retrospective studies. Our study has some potential confounding variables including the tailoring of therapy to each patient, underlying differences in cause of uveitis, severity of disease, duration of the disease, and concomitant immunomodulatory treatment. It was conducted in a single geographic area and, therefore, may not be generalizable to other populations. However, this is a pilot study, and although small in number of participants, it is the first to address the impact of dosage in the treatment of uveitis in the pediatric population. We also acknowledge that the improvement in IOP noted in MD and HD groups may not be an effect of INF alone and the concomitant weaning of steroids may have played a role. 

The data from our cohort do not suggest optimal dosing, as patients in all three groups had significant failure rates, and the groups were not ideally comparable as disease duration, severity, and uveitis types differed in each group. In this study cohort, risk factors for a poor prognosis included panuveitis, posterior uveitis, disease chronicity, prolonged topical and systemic steroid use, and prior DMARD failures. Nevertheless, despite using higher doses no serious side effects were noted during the observation period, suggesting that INF even at higher doses might be safe in the short term. Our study emphasizes the necessity of well-controlled trials to address the issue of standardization of dosing the optimal timing and duration of INF therapy in pediatric uveitis.

## Figures and Tables

**Table 1:** Demographic and treatment characteristics of children treated with high dose (HD), moderate dose (MD), and low dose (LD) Infliximab.

	No.	Age (yr)	Gender	Ethnicity	Ocular diagnosis	Systemic diagnosis	Age at diagnosis (yr)	Age at starting treatment (yr)	Failed meds before INF	Duration of treatment with Steroids before INF (mo)
HD	1	20	F	H	Anterior	ERA	15	20	MTX, PRED, TPRED	56
	2	7	F	H	Anterior	Poly JIA	5	7	MTX, PRED, TPRED	22
	3	9	F	H	Anterior	Idiopathic	7	9	MTX, TPRED, PRED, MMF	20
	4	11	F	H	Anterior	CNO	9	11	MTX, TPRED, PRED	22
	5	16	M	C	Intermediate	Idiopathic	8	16	MTX, PRED, TPRED	80
	6	18	F	H	Posterior	Idiopathic	13	18	MTX, TPRED	56
	7	6	F	H	Panuveitis	Idiopathic	5	6	MTX, TPRED	10
	8	7	M	C	Panuveitis	VKH	5.5	7	MTX, TPRED	12
	9	15	F	H	Panuveitis	VKH	10	15	MTX, PRED, TPRED	56

MD	10	4	M	C	Anterior	PSA	3	4	MTX/TPRED	10
	11	13	M	C	Anterior	ERA	11.5	13	MTX/TPRED	12
	12	13	F	C	Anterior	Oligo JIA	8	11	MTX, TPRED	36
	13	2	F	H	Anterior	Oligo JIA	1	2	MTX, TPRED	10
	14	6	M	C	Anterior	Oligo JIA	3.8	6	MTX, TPRED, PRED	20
	15	9	M	C	Anterior	Idiopathic	6	9	MTX,TPRED	28
	16	5	M	C	Intermediate	Idiopathic	3	5	MTX, TPRED	20
	17	5	M	C	Posterior	Idiopathic	3.5	5	MTX, TPRED	16
	18	11	F	H	Posterior	Idiopathic	8	11	MTX, TPRED, PRED, AZA	20
	19	8	F	H	Posterior	Idiopathic	6.5	8	MTX, TPRED, PRED	12
	20	18	F	H	Posterior	Sarcoidosis	13	18	MTX,TPRED	50

MD	21	13	M	C	Panuveitis	Idiopathic	9	13	MTX, TPRED, PRED	46
	22	15	F	H	Panuveitis	Idiopathic	13	15	MTX, TPRED	20
	23	14	M	C	Panuveitis	Idiopathic	12	14	MTX, TPRED, PRED	22
	24	15	M	C	Panuveitis	Idiopathic	13	15	MTX, TPRED	20
	25	16	F	H	Panuveitis	Idiopathic	13	16	MTX, TPRED	28
	26	11	M	C	Panuveitis	Idiopathic	8	11	MTX	28
	27	16	F	H	Panuveitis	Idiopathic	14	16	MTX/TPRED	22
	28	14	M	C	Panuveitis	Blau syndrome	9	14	MTX, TPRED, PRED	48

LD	29	12	F	H	Anterior	Poly JIA	10.5	12	MTX, PRED, TPRED	8
	30	8	F	H	Anterior	ERA	6	8	MTX/CYS/TPRED	22
	31	10	F	C	Anterior	Poly JIA	8.5	10	MTX/TPRED	8
	32	12	M	A	Anterior	Oligo JIA	10.5	12	MTX/TPRED	6
	33	11	M	C	Anterior	Oligo JIA	9.5	11	MTX/TPRED	8
	34	12	F	C	Anterior	Oligo JIA	8.5	12	MTX/TPRED	12

HD-high dose; MD-moderate dose; LD-low dose; M-male; F-female; H-hispanic; C-caucasian; A-african american; ERA-enthesitis-related arthritis; Poly JIA-polyarticular juvenile idiopathic arthritis; oligo JIA-oligoarticular juvenile idiopathic arthritis; MTX-methotrexate; PRED-prednisone; AZA-azathioprine; TPRED-topical prednisone; CYS-cyclosporine; MMF-mycophenolate mofetil; PSA-psoriatic arthritis; VKH-vogt-koyanagi-harada syndrome; CNO-chronic noninfectious osteomyelitis; Yr-year; Mo-month.

**Table 2:** Comparison of ocular findings of patients treated with high-dose, moderate-dose, and low-dose infliximab at baseline examination and at one-year followup.

		ACD				Flare				Visual acuity				IOP			
	Patient	Baseline		12 month		Baseline		12 month		Baseline		12 month		Baseline		12 month	
		OS	OD	OS	OD	OS	OD	OS	OD	OS	OD	OS	OD	OS	OD	OS	OD
																	
HD	1	2+	2+	0	0	3+	1+	1+	0	HM	8ft/200	HM	1ft/200	H	H	H	N
	2	3+	0	1+	0	3+	2+	1+	0	HM	20/20	HM	20/20	H	N	H	N
	3	2+	2+	2+	1+	1+	1+	1+	0	20/60	20/30	20/100	20/60	H	H	N	N
	4	2+	2+	1+	1+	3+	2+	0	0	20/800	20/800	20/20	20/70	H	H	N	N
	5	2+	2+	0	0	0	1+	0	0	20/400	20/20	20/20	20/20	H	N	H	N
	6	2+	2+	2+	2+	3+	3+	1+	1+	20/125	5ft/400	20/125	HM	N	H	N	H
	7	2+	1+	0	0	1+	2+	0	0	LP	20/200	LP	20/80	H	N	H	N
	8	3+	3+	0	0	3+	3+	0	0	20/400	20/200	20/40	20/40	H	H	N	N
	9	1+	2+	2+	2+	2+	2+	1+	1+	20/300	20/40	20/200	20/100	H	N	N	N

MD	10	2+	2+	0	0	2+	2+	0	0	20/60	20/40	20/40	20/40	N	N	N	N
	11	1+	1+	0	0	2+	1+	0	0	20/400	20/30	20/50	20/20	H	N	H	N
	12	1+	1+	0	0	1+	1+	0	0	20/30	20/40	20/30	20/30	N	H	N	N
	13	3+	2+	1+	1+	2+	2+	1+	1+	20/25	20/25	20/20	20/20	H	N	N	N
	14	1+	1+	0	0	2+	2+	0	0	20/25	20/25	20/20	20/25	H	N	N	N
	15	2+	2+	0	0	1+	1+	0	0	20/30	20/30	20/20	20/20	N	N	N	N
	16	2+	2+	2+	1+	2+	3+	0	0	20/25	20/40	20/20	20/60	H	N	N	N
	17	2+	2+	1+	1+	1+	1+	0	0	20/100	20/20	20/60	20/20	N	N	N	N
	18	2+	2+	1+	1+	2+	2+	0	1+	20/100	20/200	20/60	20/400	N	N	N	H
	19	2+	2+	1+	1+	1+	1+	0	0	20/200	20/200	20/200	20/200	N	N	N	N
	20	1+	1+	1+	1+	1+	1+	0	0	20/80	LP	20/40	LP	N	H	N	H
	21	2+	2+	0	0	2+	3+	0	0	20/20	20/400	20/40	20/200	N	N	N	N
	22	2+	2+	0	0	2+	2+	0	0	20/200	20/200	20/40	20/40	H	N	N	N
	23	3+	3+	0	0	2+	2+	0	0	20/20	20/40	20/20	20/20	N	N	N	N
	24	NA	2+	NA	0	0	1+	0	0	20/40	20/200	20/40	20/40	N	N	N	N
	25	1+	1+	0	0	2+	1+	0	0	20/60	20/40	20/20	20/20	N	N	N	N
	26	2+	2+	0	0	2+	2+	0	0	20/20	20/40	20/20	20/20	N	N	N	N
	27	1+	1+	0	0	2+	2+	0	0	20/80	20/20	20/60	20/20	N	N	N	N
	28	2+	2+	0	0	1+	1+	0	1+	20/20	20/20	20/20	20/20	N	N	N	N

LD	29	2+	1+	0	0	3+	2+	0	0	20/100	20/20	20/20	20/20	N	N	N	N
	30	1+	1+	0	0	0	1+	0	0	20/20	20/80	20/20	20/30	N	N	N	N
	31	2+	2+	0	0	2+	2+	0	0	20/40	20/30	20/80	20/30	N	N	N	N
	32	2+	1+	0	0	1+	1+	0	0	20/20	20/20	20/20	20/20	N	N	N	N
	33	1+	2+	0	0	1+	2+	0	0	20/20	20/20	20/20	20/20	N	N	N	N
	34	1+	2+	0	0	1+	2+	0	0	20/20	20/20	20/20	20/20	N	N	N	N

HD-high dose; MD-moderate dose; LD-low dose; ACD-anterior chamber cell density; IOP-intraocular pressure; LP-light perception; HM-hand movement; N-normal; H-high; Ft-feet; OS-ocular sinister; OD-oculus dexter.

## Abbreviations

INF: Infliximab
ETN: Etanercept
SUN: Standardization of uveitis nomenclature
JIA: Juvenile idiopathic arthritis
TNF: Tumor necrosis factor
LD: Low dose
MD: Moderate dose
HD: High dose
HACA: Human antichimeric antibody
VEGF: Vascular endothelial growth factor
OD: Oculus dexter (right eye)
OS: Oculus sinister (left eye)
OU: Oculi utrique (both eyes).
